# Recent Advances in Endometriosis Pathophysiology and Pharmacological Treatment

**DOI:** 10.3390/ijms25126575

**Published:** 2024-06-14

**Authors:** Alexandra Perricos, René Wenzl

**Affiliations:** Department of Obstetrics and Gynecology, Medical University of Vienna, 1090 Vienna, Austria; alexandra.perricos@meduniwien.ac.at

Endometriosis, affecting an estimated 10% of women of reproductive age [1], remains an enigmatic disease. Over the past decades, researchers have tried to determine the exact pathophysiology of this disease in the hope of not only a better understanding but also the expansion of targeted pharmacological therapies. The intention behind this Special Issue was to highlight these important attempts to achieve a better understanding of endometriosis.

Several authors addressed the fundamental questions surrounding the pathogenesis of endometriosis. Lamceva et al. provided an extensive review of the main theories on the pathogenesis of endometriosis, discussing retrograde menstruation, benign metastasis, immune dysregulation, and coelomic metaplasia, underscoring the complexity of the disease and the interplay of multiple factors that contribute to its development and progression [2]. The widespread theory of retrograde menstruation, published by Sampson in 1927 [3], suggested that menstrual blood consisting of vital endometrial cells flows backward through the fallopian tubes into the pelvic cavity, where these endometrial cells then implant and proliferate. This theory does not satisfyingly explain the occurrence of this disease however, considering that ca. 90% of women demonstrate retrograde menstruation. Furthermore, while retrograde menstruation might contribute to endometriotic lesions in the pelvic/abdominal cavity, it does not explain the extra-pelvic lesions. Although these extra-pelvic lesions, e.g., in the lung, lymph nodes, and abdominal wall, would support the theory of benign metastasis, the ability of endometriotic cells to enter and exit lymphatic and blood vessels has recently been called into question [4]. Coelomic metaplasia, a theory first discussed in 1942 [5], could offer another possible explanation for the development of these lesions that reach far outside the pelvis.

Immune dysregulation is a crucial factor in the pathogenesis of endometriosis. The immune system’s inability to completely recognize and destroy ectopic lesions allows these cells to survive and proliferate [6]. The involvement of various immune cells such as macrophages, natural killer cells, and leukocytes highlights the complex interaction between the immune system and endometriotic lesions [7,8,9]. Furthermore, there seem to be differences in the importance of the immune response within the different endometriosis subtypes. Not only have differences in systemic as well as local immune profiles been described between patients with endometriosis and controls [10], but in patients with deep-infiltrating endometriosis (DIE), different local immune environments have been observed when compared to patients with other endometriosis subtypes. This might be due to the singular characteristics of DIE lesions, which are not unlike those of cancerous cells, showing increased proliferation and deeper infiltration into the surrounding tissue. Next to the unique inflammatory environment, the important role of oxidative stress has also been described in numerous studies. Oxidative stress, which partly results from an imbalance of Reactive Oxygen Species (ROS) and antioxidants, is known to cause the proliferation of endometriotic cells and has been described as increased not only in the local but also in the systemic environment of women with endometriosis compared to controls [11].

A detailed evaluation and insight into the local and systemic environment of this disease is important not only in order to achieve a better understanding of its pathophysiology but also to enable a non-invasive diagnosis in the future. While most endometriosis subtypes such as DIE, ovarian endometriosis, and adenomyosis can be diagnosed through imaging techniques such as transvaginal sonography or Magnetic Resonance Imaging [12,13], peritoneal lesions can still only be diagnosed and consecutively treated through surgery. Considering the risks associated with any surgical procedure and the long delay between the onset of symptoms and the diagnosis [14], the crucial importance of the search for adequate serum biomarkers, perhaps in the shape of multiplex panels with high sensitivity and specificity, is underlined.

Endometriosis is known to be driven by a hormonal imbalance in terms of estrogen dominance and progesterone resistance [15]. While current guidelines recommend hormonal therapies such as combined oral contraceptives, gestagens, or GnRH agonists and antagonists [16], researchers constantly search for hormone-free alternatives, driven by a clear trend away from hormones in this particular patient collective, as well as lacking therapeutic options for women trying to conceive, yet suffering from severe endometriosis-associated symptoms, women for whom hormonal therapies are not adequate due to their contraceptive effect. While some studies evaluate lifestyle changes [17], nutritional supplements, or complementary medicine as alternative treatment options, others, such as Guan et al., search for medications that primarily exert their efficacy by providing pain relief. This research group analyzed the effect of opioids on endometriosis-associated pain and found their use in this group of patients limited, due to an increased risk of addiction resulting from chronic opioid use in patients with endometriosis, although they suggested a possible positive outlook regarding multi-mechanistic opioids for the treatment of endometriosis-associated pain, due to their safer profile [18].

The future of endometriosis research lies in a multidisciplinary approach that integrates insights from immunology, genetics, molecular biology, and environmental sciences. Continued collaboration among researchers, clinicians, and patients is essential to uncover the full spectrum of endometriosis pathogenesis and to develop comprehensive management strategies. Promising areas of research include the identification of novel biomarkers for early diagnosis and the development of targeted therapies that both address specific molecular pathways. As our understanding of the disease evolves, so too will the opportunities for innovative treatments that improve the quality of life for those affected by endometriosis.

This Special Issue of the International Journal of Molecular Sciences brings together research that will enhance our understanding of endometriosis. The contributions of these studies not only elucidate the underlying mechanisms of the disease but also pave the way for innovative diagnostic and therapeutic strategies. Continued research and collaboration in this field are essential to address the unmet needs of patients with endometriosis and to improve their quality of life. We extend our gratitude to all the authors for their valuable contributions and to the reviewers for their critical evaluations. We hope this Special Issue serves as a significant resource for researchers and clinicians involved in the fight against endometriosis.
